# Research on Detection Mechanism of Weld Defects of Carbon Steel Plate Based on Orthogonal Axial Eddy Current Probe

**DOI:** 10.3390/s20195515

**Published:** 2020-09-26

**Authors:** Linnan Huang, Chunhui Liao, Xiaochun Song, Tao Chen, Xu Zhang, Zhiyang Deng

**Affiliations:** Hubei Key Laboratory of Modern Manufacturing Quantity Engineering, School of Mechanical Engineering, Hubei University of Technology, Wuhan 430068, China; hblhkhln@163.com (L.H.); songxc@mail.hbut.edu.cn (X.S.); chentao@hnu.edu.cn (T.C.); zhangxu@mail.hbut.edu.cn (X.Z.); dzy@hust.edu.cn (Z.D.)

**Keywords:** eddy current testing, carbon steel weld, orthogonal axial probe, lift-off effect

## Abstract

The uneven surface of the weld seam makes eddy current testing more susceptible to the lift-off effect of the probe. Therefore, the defect of carbon steel plate welds has always been a difficult problem in eddy current testing. This study aimed to design a new type of eddy current orthogonal axial probe and establish the finite element simulation model of the probe. The effect of the probe structure, coil turns, and coil size on the detection sensitivity was simulated. Further, a designed orthogonal axial probe was used to conduct a systematic experiment on the weld of carbon steel specimens, and the 0.2 mm width and 1 mm depth of weld defects of carbon steel plates were effectively detected. The experimental results showed that the new orthogonal axial eddy current probe effectively suppressed the unevenness effect of the weld surface on the lift-off effect during the detection process.

## 1. Introduction

Carbon steel materials were widely used in nuclear power generation, oil and gas pipeline transportation, machinery manufacturing, and transportation. Carbon steel plates were mainly used for welding as components of pressure-bearing equipment [1]. Due to long-term high temperature, high pressure, causticity, and other adverse changes in the working environment, carbon steel welds often have cracks, corrosion, small holes, and other defects, resulting in early damage to the equipment and even harm to human life [2]. Therefore, regular inspection of the weld area of the carbon steel plate is mandatory. During the eddy current testing process, contacting the workpiece or requiring a coupling agent is unnecessary. The eddy current testing can be done in a high-temperature environment, and, at the same time, the testing has a high sensitivity for surface defect detection. In the process of detecting the weld seam, the geometry of the weld seam and bead varies, and the surface is uneven, reducing the signal-to-noise ratio of the test and affecting the accuracy of the test [3,4]. Researchers at home and abroad have conducted some studies to resolve the problem of difficult inspection of the weld seam. Haixia et al. [5] analyzed the changes in induced eddy current and perturbed magnetic field in the test specimen and gave the rule of the detecting the change in signal with the lift-off under different conditions. Guiyun et al. [6] used two reference signals for the lift-off effect of eddy current detection, calculated the differential signaling in two stages, and achieved great success in the case of metal loss and subsurface grooves. In view of the lift-off effect of eddy current detection, a Hoshi probe, as a fundamental uniform eddy current (UEC) probe, was invented by Hoshikawa. It consists of a tangential rectangular excitation coil and a circular detection coil, in which the circular coil is placed under the rectangular coil. A Hoshi probe was designed to detect flaws such as cracks on the weld zone having uneven surface a nonmagnetic material [7,8,9]. In Reperianto et al. [10] the concept of a uniform eddy current is introduced, and some used uniform eddy current probes are introduced on the basis of uniform eddy current. The application technology showed that the probe design had a decent effect on the weld seam. Damhuji Rifai et al. [11] designed the UEC probe with a giant magnetoresistance (GMR) detector. The probe consists of one tangential rectangular excitation coil and a GMR detector. The probe can detect deep defects at frequencies below 1 kHz. Kral et al. [12] described the lift-off effect well by establishing a linear transformer to understand the meaning of the physical foundation behind the pulsed eddy current measurement. Santos et al. [13,14] designed a planar differential coil to scan the defects of aluminum plate welds, proving that the probe could easily scan the welding defects of artificial friction spots. The probe had the reliability of detecting deep defects at lower frequencies. The recent probe design that uses the UEC principle was called the IOnic probe. The probe consists of one tangential rectangular excitation coil and two semicircular coils are placed differential and symmetrically as a detector. A probe can be used to detect weld defects and fine flaws on the plate. However, the detection coils on both sides of the probe need to be made with high precision [15]. Another probe, butterfly probe, was designed based on UEC. The probe is excited by a pair of symmetrical rectangular plane coils. A circular coil is placed under the two excitation coils for detection. The coil is excited by a phase difference of 90°. The probe has high sensitivity in detecting plate defects, but the detection of weld defects is not as effective as traditional UEC probe [16].

The traditional orthogonal probe is placed by two rectangular coils standing crosswise. At present, because it has better suppression of lift-off effect than conventional eddy current probes, most researchers still use traditional orthogonal probes to detect weld defects. However, the distribution of the eddy current generated by it is mainly on the surface of the probe, and the uniform eddy current has the characteristics of self-nulling [7]. As a result, the sensitivity and resolution of the probe scan for weld defects are not high enough. Therefore, on the basis of the UEC, we designed the orthogonal axial probe and an in-depth investigation of the eddy current lift-off effect caused by uneven seam weld surface was carried out. The parameters affecting the sensitivity of the probe are also studied. The probe cannot only suppress the eddy current lift-off effect but also maintain high sensitivity when detecting welds. By changing the direction of the eddy current field generated by the probe, the change in the eddy current impedance when the probe passed through the weld seam area without defects was found to be smaller than that of the conventional eddy current probe, and has stronger sensitivity than UEC probe. Finally, the feasibility of the method is proved by simulation and experiment research.

## 2. Simulation Optimization and Structure Design of the Probe

The lift-off effect caused by the unevenness of the weld seam surface has always been a drawback due to the high sensitivity of eddy current testing to lift-off, which the eddy current testing needs to overcome. The eddy current field generated by the conventional eddy current probe is perpendicular to the surface of the measured object. When the probe is shaken or scanned in the weld seam area, the coil impedance changes due to the up and down fluctuations of the probe, so that the detection signal obtained by the probe responds at the nondefective position as well. The signal-to-noise ratio of the detected signal also becomes worse [17]. Based on the principle of eddy current detection, this study found through theoretical analysis that when the relative motion direction of the eddy current field and the defect of the tested part changed, the eddy current field was more uniform than that generated by the conventional probe. The detection coil was only sensitive to the eddy current component parallel to the surface of the measured object, which reduced the impedance change of the eddy current probe caused by the fluctuation during the probe scanning process. It also had a decent suppression effect on the lift-off effect caused by the uneven surface of the weld seam.

**Figure 1:**
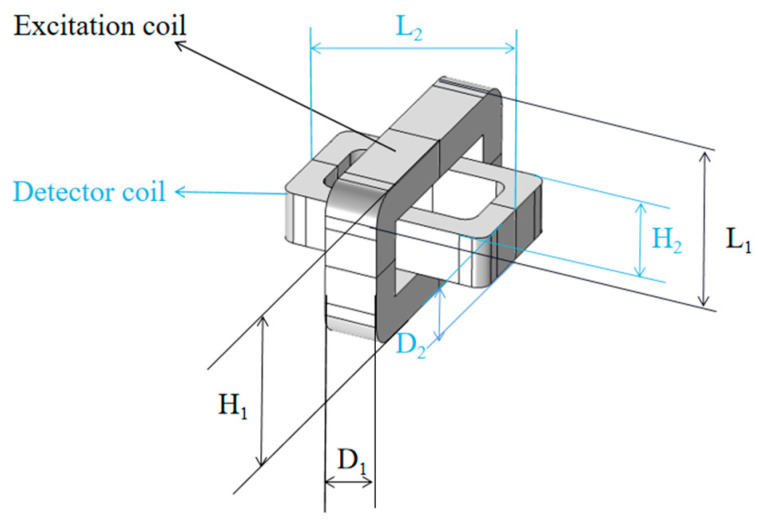
Orthogonal axial probe structure.

According to this principle, the author designed a kind of orthogonal axial probe that could form a tangential uniform eddy current in this study. The probe was composed of two oblong rectangular coils placed orthogonal to each other, as seen in Figure 1. The excitation coil was placed perpendicular to the surface, and the detection coil was placed axially at the center of the excitation coil, and L is Length, H is Width, and D is Height. Compared with the conventional probe, the magnetic field generated by the eddy current of this probe was parallel to the surface of the test specimen. When passing through the uneven weld seam surface, the direction of the eddy current changed little, and the output signal change value caused by the lift-off was small [18]. Moreover, the eddy current field generated by the tangential excitation coil was more uniform, making the impedance change sensitivity caused by the lift-off effect less than that of the conventional eddy current probe. When passing through the weld seam area, except for the eddy current disturbance to the defect location, the lift-off effect produced by the remaining weld area had no such disturbance. The detection signal had a high signal-to-noise ratio, obvious defect characteristic signal, and high sensitivity, which, to a certain extent, suppressed the eddy current lift-off effect caused by the unevenness of the weld seam.

**Figure 2:**
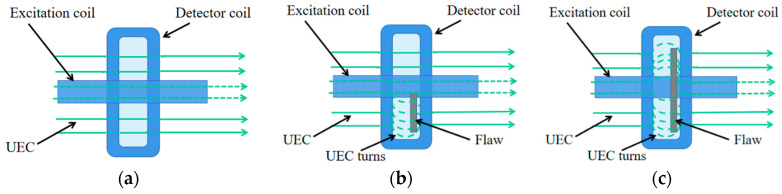
The eddy current flows under an orthogonal axial prob: (**a**) without a flaw; (**b**) with an asymmetric flaw; (**c**) with a flaw in the lower middle of the probe.

Figure 2 shows the UEC distribution when using the orthogonal axial prob. When the probe is detected without defects, the detector coil does not generate the electromotive force (EMF). Furthermore, if the probe is scanning the surface similar to the weld, the lift-off value changes, and the EMF will still be zero value since there is no change of direction of the eddy current flow [10]. As shown in Figure 2b. When the flaw is located at the edge of the detection coil, it causes the current of the detection coil to change and produces an output, as shown in Figure 2c. When the flaw is in the middle of the probe, since the electromotive force of the same amplitude is generated on both sides, but the polarities are opposite, this is the output of the detection coil is zero, and this phenomenon is called self-nulling.

### 2.1. Simulation Model Establishment

In this study, COMSOL Multiphysics was used to establish a carbon steel weld seam defect detection model so as to study the suppression effect of the orthogonal axial probe on the unevenness of the weld seam area and the directionality of the eddy current field generated by the probe on the carbon steel plate surface. The simulation model consists of a circular air domain, excitation coil and detection coil, and carbon steel plate, and the parameters and materials are shown in Table 1 and Table 2. The simulation uses the infinite element domain in the setting of the physical field boundary conditions to realize the approximate simulation of the electromagnetic field and uses the impedance boundary conditions for the carbon steel plate specimens, which reduces the calculation difficulty and realizes the analysis of the characteristics of the eddy current. Physics is used to control meshing, and the mesh size is refined. The physical field is selected as the magnetic field under the AC/DC module, and researched and solved in the frequency domain. The above simulation analysis is based on the magnetic vector potential method. In this method, the magnetic scalar potential *Ω* and current vector potential *T* are used to represent the electromagnetic field [16]. The equation involving the method are expressed by Ampere’s law, Faraday’s law, and constitutive relation:

(1) $$\nabla \times \vec{E}=-\frac{\partial \vec{B}}{\partial t}$$

(2) $$\vec{B}=\mu \vec{H}$$

(3) $$\vec{J}+\varepsilon \frac{\partial \vec{E}}{\partial t}=\nabla \times \vec{H}$$

(4) $$\vec{E}={\left(\sigma +\varepsilon \frac{\partial }{\partial t}\right)}^{-1}\cdot \vec{J}$$

where the various quantities are defined as

$\vec{E}$: Electric field intensity (V/m)
$\vec{H}$: Magnetic field intensity (A/m)
$\vec{B}$: Magnetic flux density (T)
$\vec{J}$: Current density (A/m^2^)
t: Time (s)
ε: Permittivity (F/m)
μ: Magnetic permeability (H/m)
σ: Conductivity (S/m)

Substituting Equation (4) into Equation (1) results in the following equation:

(5) $$\nabla \times \left[{\left(\sigma +\varepsilon \frac{\partial }{\partial t}\right)}^{-1}\cdot \vec{J}\right]+\frac{\partial \vec{B}}{\partial t}=0$$

Substituting Equation (2) into Equation (5) After eliminating $\vec{B}$:

(6) $$\nabla \times \left[{\left(\sigma +\varepsilon \frac{\partial }{\partial t}\right)}^{-1}\cdot \vec{J}\right]+\mu \frac{\partial \vec{H}}{\partial t}=0$$

Finally, by substituting Equations (3) into Equation (6), the governing equations are obtained:

(7) $$\nabla \times \left[{\left(\sigma +\varepsilon \frac{\partial }{\partial t}\right)}^{-1}\cdot \nabla \times \vec{H}\right]+\mu \frac{\partial \vec{H}}{\partial t}=0$$

It can be seen from Formula (4)

(8) $$\vec{J}=\sigma \vec{E}+\varepsilon \frac{\partial \vec{E}}{\partial t}$$

The software is used together with Equations (7) and (8). 

The simulation uses the infinite element domain to realize the approximate simulation of the electromagnetic field. The impedance boundary condition is adopted in the physical field boundary condition setting, which reduces the calculation difficulty and realizes the approximate distribution characteristic analysis of the eddy current. 

Through simulation, it was found that when the lift-off value is 0.5 mm, the sensitivity of the detection coil increased with the number of turns in a certain range. However, when the number of turns continued to increase, it instead affected the probe sensitivity because the probe impedance and heat generation increased [17]. Therefore, the probe turns were set as 200 turns through the previous relevant tests. At this time, the probe sensitivity is better. 

The excitation coil parameters are L_1_ = 7 mm, H_1_ = 5 mm, and D_1_ = 2 mm. The detection coil parameters are L_2_ = 5 mm, H_2_ = 3 mm, and D_2_ = 2 mm. Models were made by COMSOL Multiphysics to study the effect of the orthogonal axial probe on the welding seam area scan so as to suppress the lift-off effect. Moreover, an orthogonal axial probe was constructed on the defect-free carbon steel plate specimen (*l* = 40 mm, *w* = 40 mm), to carry out the physics simulation analysis and comparison of a conventional circular eddy current probe of a similar size. First, place the conventional circular probe and orthogonal axial probe above the carbon steel plate with a lift-off value of 0.5 mm. Use 5 V AC voltage and 100 kHz frequency sine wave to excite the excitation coil. After solving, set up and extract the modulus of current density generated by the two probes on the surface of the carbon steel plate. 

**Figure 3:**
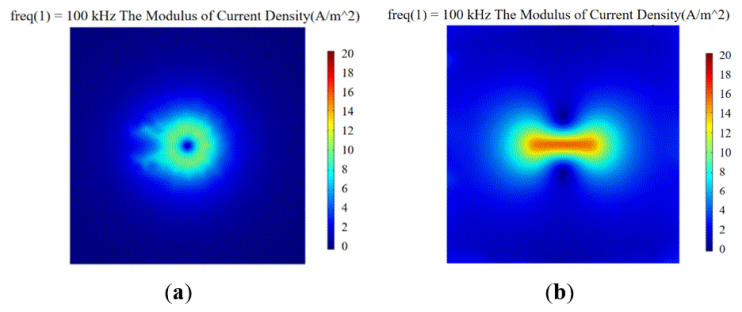
Distribution diagram of surface current density modulus of different probes: (**a**) conventional circular probe; (**b**) orthogonal axial probe.

Figure 3a shows the distribution diagram of the surface current density modulus produced by the conventional circular probe, and Figure 3b shows the distribution diagram of the current density modulus produced by the orthogonal axial probe. The numerical value of the intensity distribution of the eddy current field generated by the probe was obtained by dividing the contour of the simulation. Compared with the current density mode generated by the conventional circular eddy current probe, the orthogonal axial probe was placed on the side, so that the generated eddy current field passed tangentially parallel through the surface of the carbon steel plate. The intensity distribution of the eddy current field was uniform, while the amplitude of the eddy current field produced by the conventional circular eddy current probe differed much. The difference in the magnitude of the eddy current field generated by the probe was extracted, calculated by Formula (9).

(9) $$\xi =\frac{{\vec{J}}_{emax}-{\vec{J}}_{emin}}{\frac{{\vec{J}}_{emax}+{\vec{J}}_{emin}}{2}}\times 100\%$$

where ξ is induced eddy current fluctuation difference. ${\vec{J}}_{emax}$ is maximum induced eddy current density (A/m^2^). ${\vec{J}}_{emin}$ is minimum induced eddy current density (A/m^2^).

The difference in the magnitude of the eddy current field generated by the probe was extracted, and the center amplitude difference of the eddy current field of the conventional circular probe was about 56% larger than that of the orthogonal axial probe. The orthogonal eddy current field distribution of the orthogonal axial probe passing through the defect-free flat plate was found to be more uniform than that of the conventional circular probe, and the eddy current field intensity uniformity was better.

**Figure 4:**
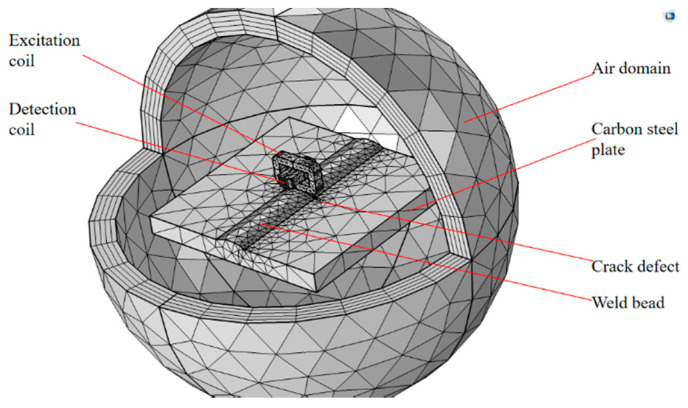
Simulation model of weld defect scanning.

Based on the data in Table 1, the author establishes a simulation model to compare the uniformity of the eddy current field distribution on the uneven weld seam defects between the conventional circular probe and the orthogonal axial probe. As shown in Figure 4, the model uses a surface with a bump to simulate a real, uneven weld surface, the crack defect is below the probe, so as to simulate the scene when the probe is scanning the uneven surface of the weld 

**Figure 5:**
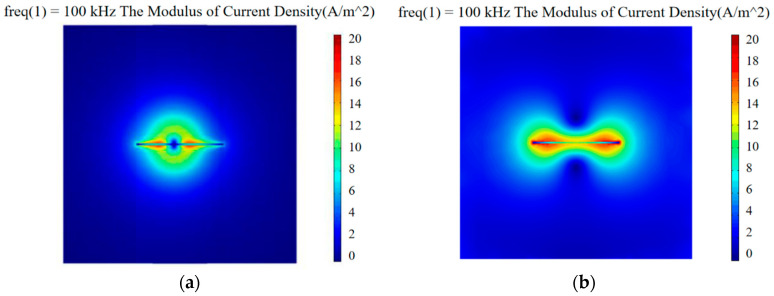
Distribution diagram of surface current density modulus of different probes with defects: (**a**) conventional circular probe; (**b**) orthogonal axial probe.

In order to compare with the simulation results in Figure 3, we placed the probe in Figure 3 on the carbon steel plate with welding crack defects under the same excitation conditions and model conditions, and extracted the modulus of current density on welding crack defects. In Figure 5. When scanning the uneven weld seam defects, the eddy current field generated by the orthogonal axial probe was far more uniform than that generated by the conventional circular probe. The difference in amplitude intensity of the eddy current field was obtained by dividing the contour. The amplitude difference in the eddy current field intensity of the conventional circular probe was calculated to be about 43% larger than that of the orthogonal axial probe, indicating that the eddy current uniformity of the orthogonal axial probe was also better than that of the conventional circular eddy current probe in the process of scanning the uneven weld seam defects.

In addition, the high permeability of the carbon steel plate resulted in the lower penetration depth of the eddy current. Part of the eddy current field generated by the orthogonal axial probe was distributed on the surface of the carbon steel plate [19], and the rest of the eddy current field passed tangentially through the weld seam surface of the carbon steel plate, making the distribution of the eddy current field in the detection area uniform. Furthermore, the probe had the effect of self-compensating the reverse eddy current field when detecting uneven surfaces because of its symmetrical distribution, so that it could better suppress the lift-off effect caused by the weld seam unevenness when scanning the uneven weld seam area.

### 2.2. The Influence of Coil Width on Output Signal

Probe sensitivity is important in judging how good the detection effect is. Many factors influence the sensitivity of the probe, including the size of the coil, number of turns, distance between the coils, distance of the probe lift-off, and size of the excitation signal [20,21]. This study compared the absolute average value of the amplitude obtained after the probe scans for defects to explore the detection sensitivity of the probe, in Formula (10).

(10) $$\tau =\frac{\left(|\sigma_{\min}|+|\sigma_{\max}|\right)}{2}$$

**Figure 6:**
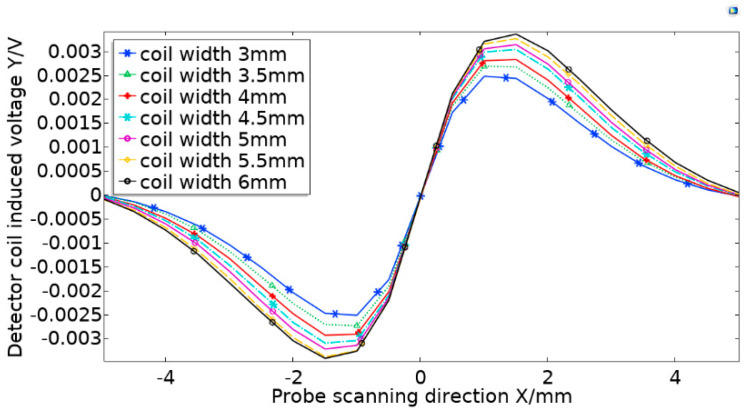
Detecting coil width change simulation scan waveform diagram.

The values of $\sigma_{\max}$ and $\sigma_{\min}$ are the maximum amplitude and minimum amplitude during scanning. Here are the maximum and minimum values of the simulated waveform during the parametric sweep in the simulation, as shown in Figure 6 for the highest point value and the lowest point value of each waveform. In this study, the amplitude of the simulated scanning was determined by parameterized scanning, and the absolute average values were obtained and compared. The detection coil size of the probe was the most important influencing factor for sensitivity. The coil size needs to be designed to ensure the maximum sensitivity of the probe [22]. This study used COMSOL Multiphysics software to explore the relationship between the size of the excitation coil and the sensitivity of the probe. The excitation and detection coils were initially set as L_1_ = 7 mm, H_1_ = 3 mm, D_1_ = 2 mm, and L_2_ = 7 mm, H_2_ = 3 mm, D_2_ = 2 mm to ensure that the detection coil had enough space to change the size of the excitation coil.

The influence of the detection coil width H_2_ on the probe sensitivity was studied without changing other data. Input with an excitation voltage of 5 V and an excitation frequency of 100 kHz. The size of the excitation coil remains unchanged, increase the detection coil width H_2_ from 3 mm to 6 mm at step of 0.5 mm. The weld seam crack defects dimensions, *l* = 20 mm, *w* = 0.3 mm, and *h* = 2 mm. Furthermore, scan parametrically from 5mm on the left of the defect to 5 mm on the right of the defect. The analog scan waveform is shown in Figure 5. Among them, Y/V means that the unit of Y axis is V, which represents the value of the induced voltage of the detection coil to simulate scanning defects. X/mm means that the unit of X axis is mm, which means the distance the probe moves.

**Figure 7:**
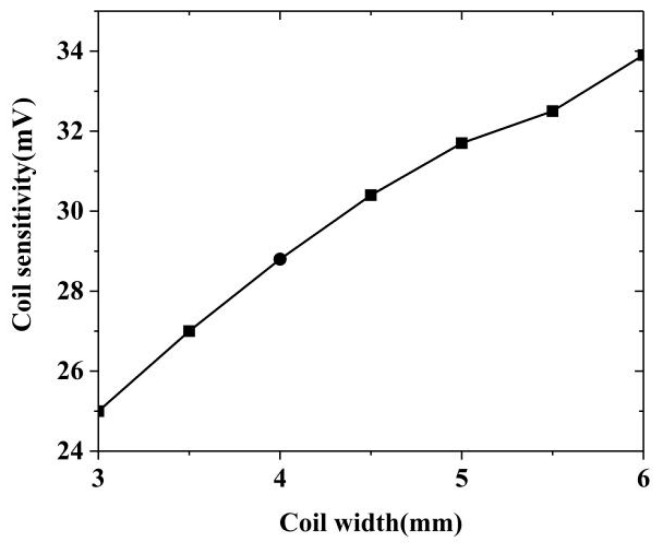
Changes in probe sensitivity caused by changes in detection coil width.

From the aforementioned Equation (9), the equivalent amplitude measured by the probe at each width and the relationship change graph between the coil width and the sensitivity could be obtained in Figure 7.

In the process of coil width change from 3 mm to 6 mm, it was found that when the probe coil width was between 3 mm and 4.5 mm, the sensitivity growth rate was approximately linear, while at 4.5 mm and 6 mm, the sensitivity still increased as the coil width increased, but the growth rate declined. In addition, if the coil width was too large, the size of the missed detection was relatively small, so it was not suitable to make an array probe. Therefore, this study selected 4.5 mm as the detection coil width, and the coil layer thickness was 1 mm, that is, the inner diameter width was 2.5 mm.

### 2.3. The Influence of Coil Length on Output Signal

**Figure 8:**
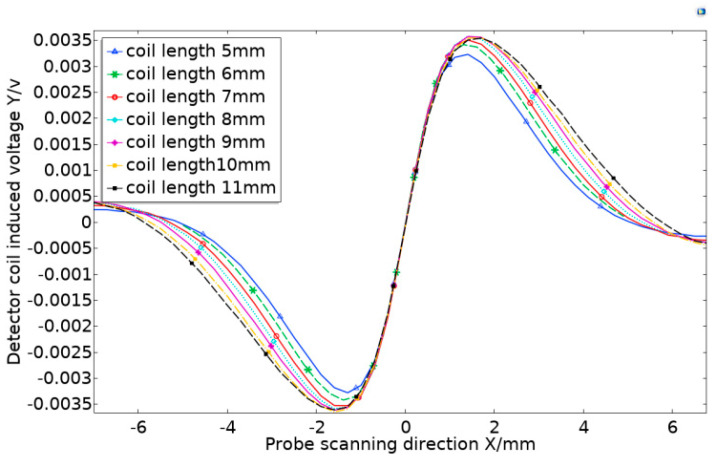
Detecting coil length change simulation scan waveform diagram.

While keeping other parameters unchanged, the inner diameter of the detection coil was set to 2.5 mm, and then the simulation analysis of the detection coil length and the detection sensitivity was carried out. With the same input conditions as above, increasing the detection coil length L_2_ from 5 mm to 11 mm at step of 1 mm, and scan parametrically from 7 mm on the left of the defect to 7 mm on the right of the defect. The analog scan waveform is shown in Figure 8.

**Figure 9:**
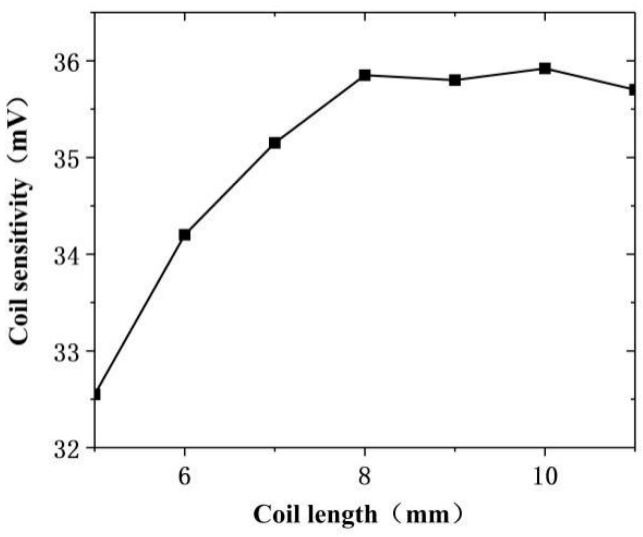
Changes in probe sensitivity caused by changes in detection coil length.

The amplitude of the probe’s simulated scanning waveform was substituted into Equation (9) to find the equivalent amplitude of the probe in each length change interval and obtain the relationship change diagram between the coil length and the sensitivity in Figure 9. 

The Figure 9 shows that when the detection coil length was 5–8 mm, the probe sensitivity increased with the increase in the coil length, and when the length was 8–11 mm, the probe sensitivity hardly changed. In addition, in actual inspection work, large-sized coils tended to miss small defects and had a low resolution. After comprehensively considering the resolution, sensitivity, missed detection, and other issues, this study selected 7 mm as the length parameter of the detection coil.

This study found that the detection coil sensitivity of the orthogonal axial probe increased with the increase in the coil width and length within a certain range. However, as the coil length continued to increase, the coil sensitivity tended to decline. Combining the simulation analysis results, the characteristics of the defects in the actual detection work, and the requirements of the coil size itself, it could be determined that when the coil width was 5 mm and the length was 7 mm, the coil sensitivity was strong, the size was suitable, the probe resolution was decent, and the overall performance was the best. Finally, the size of the orthogonal axial probe coil was selected in Table 3.

### 2.4. The Influence of Scanning Mode on Output Signal

The distribution of the eddy current field generated was not the same as that of the circular coil and was a nonaxisymmetric eddy current field since the structure of the rectangular coil was different from that of the circular coil. When performing defect detection, the magnetic field excited by the probe had a directional characteristic so that the magnetic field intensity was a value obtained by adding the magnetic field vectors in different directions [23]. The orthogonal axial probe was studied theoretically and through the magnetic field and eddy current field simulation to explore the position of the maximum magnetic field intensity during the probe scanning process.

**Figure 10:**
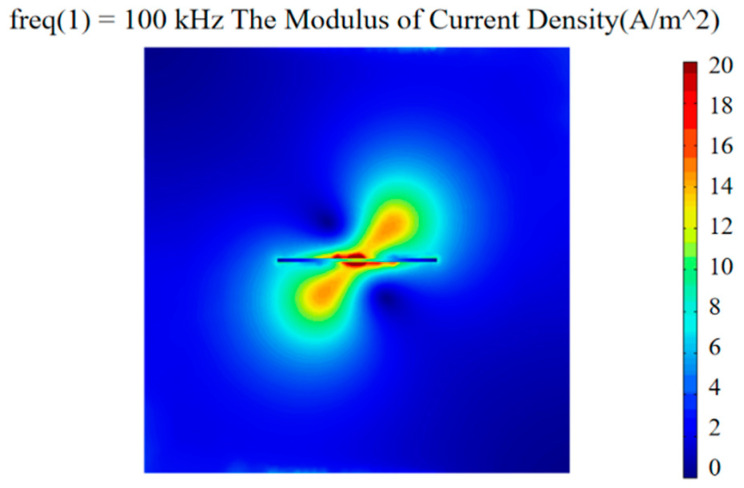
Orthogonal axial probe 45° through the defect surface current density modulus.

First, the coil size determined in Table 3 was selected to simulate the magnetic field distribution. The X-component cloud image of the probe’s magnetic field on the surface of the test piece was studied. The direction of the magnetic field component had a certain angle with the direction of the defect. Similarly, the Y-component cloud image of the magnetic field had the same angle. The position of the vector sum of the largest magnetic field component between the orthogonal axial probe and the defect during the scanning of the defect was studied by changing the direction coefficient of the magnetic field relative to the defect when the probe detected the defect, that is, changing the detection angle of the probe scanning defects. In Figure 10, it shows the surface current density mode distribution when the probe and the crack defect formed an angle of 45°. Compared with Figure 4b, the eddy current intensity of the defect position after the probe had a deflection angle of 45° was found to be higher than the eddy current intensity of the defect position without the deflection. Similarly, after simulating the random angle, the comparison chart of the eddy current intensity at the defect showed that the eddy current intensity of the defect detection when the probe had a deflection angle was greater than the eddy current intensity when the probe had no deflection. However, the intensity of the eddy current changed as the angle changed, indicating a maximum value of the magnetic field intensity. 

**Figure 11:**
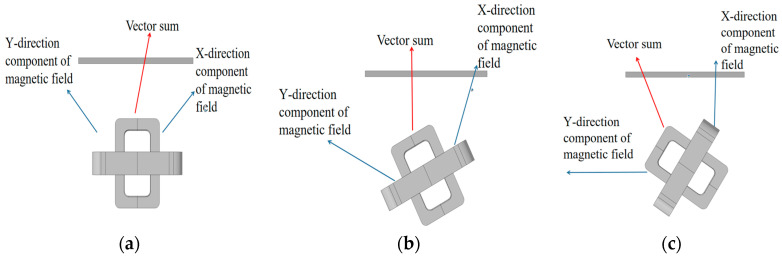
Overlay of magnetic field vector of orthogonal axial probe at different scanning angles: (**a**) probe 0° through defect; (**b**) probe 30° through defect; (**c**) probe 60° through defect.

The excitation probe was placed in parallel with the simulated defect in the simulation test software, the initial angle was set to 0°, and the step was 10° to get the best sensitivity of the probe scan and make the vector sum of the magnetic field components reach the maximum value. The variation range being 0°–90°. Figure 11 shows the top view of the model when the scanning angle of the orthogonal axial probe scanning defects was 0°, 30°, and 60°. The red line in the figure represents the vector sum of the magnetic field in the X and Y directions when the probe scanned for defects at different rotation angles.

**Figure 12:**
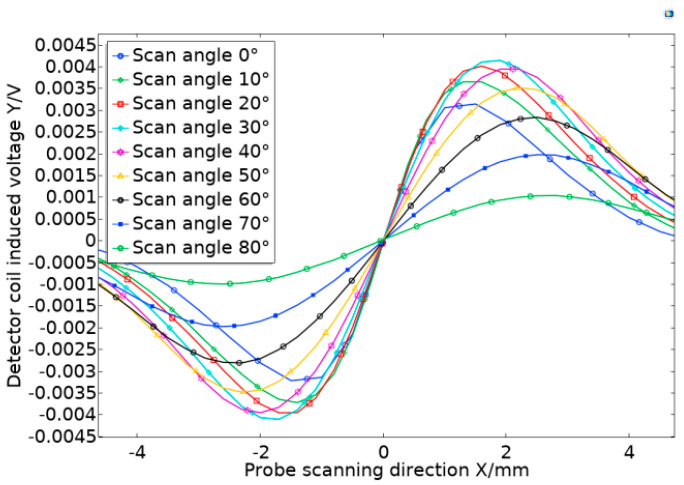
Simulated scanning waveform of orthogonal axial probe at different angles.

**Figure 13:**
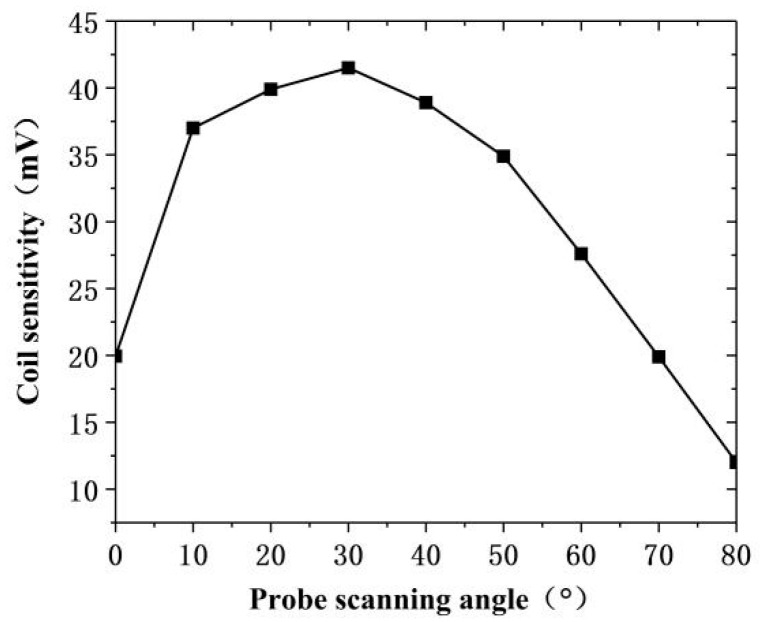
Changes in probe sensitivity caused by the probe scanning angle change.

As shown in Figure 12, the simulated scan waveform is obtained by the scan parametrically from 5 mm on the left of the defect to 5 mm on the right of the defect. Then, the sensitivity of the probe was calculated at each angle from the obtained data, and the relationship between the probe scanning angle and the probe sensitivity was obtained, in Figure 13. During the rotation of the probe, when the angle was 0°–30°, the sensitivity increased with increasing angle, while when it exceeded 30°, the probe sensitivity decreased with increasing angle. 

The simulation proved the existence of the vector sum of the magnetic field of the orthogonal axial probe and the influence of the scanning angle of the orthogonal axial probe on the sensitivity of the probe in the scanning mode. At the same time, it was found that when changing the angle between the probe and the defect, the sensitivity changed with the angle, and the sensitivity of the probe was the strongest when the deflection angle is 30°. Therefore, when we use the orthogonal axial probe, we can rotate the probe 30° for scanning inspection. 

## 3. Experimental Results and Discussion

**Figure 14:**
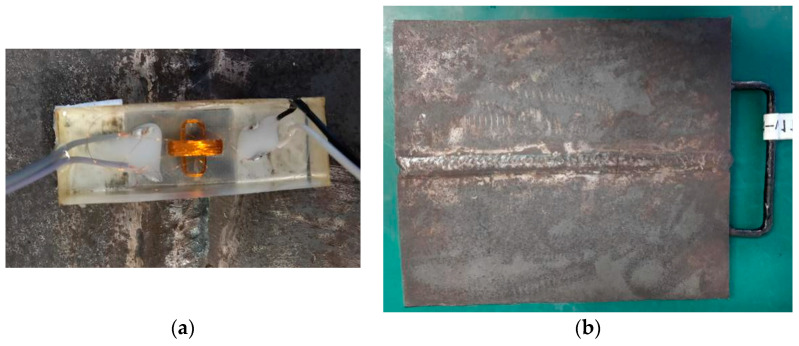
(**a**) Probe in kind; (**b**) Carbon steel plate weld defect test piece.

To verify the aforementioned theoretical and simulation test research. The size design of the orthogonal axial probe is shown in Table 3, the probe structure is shown in Figure 14a. The carbon steel test block in the Figure 14b. Because of the defect size of machine machining is too large, we use laser machining to simulate the defect direction and size of specimen in practice. The dimensions of carbon steel plate are *l* = 300 mm, *w* = 250 mm, *h* = 15 mm. The size of the three cracks (*l* × *w* × *h*) is 25 × 0.2 × 1.0 mm, 25 × 0.1 × 1.0 mm, 25 × 0.2 × 0.5 mm. Furthermore, an eddy current test bench was built.

### 3.1. Experimental Verification of the Scanning Angle of the Orthogonal Axial Probe

**Figure 15:**
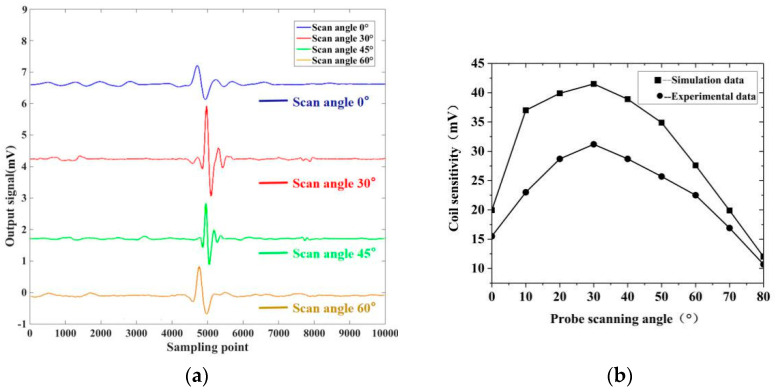
The relationship between the scanning angle of the probe and the output signal: (**a**) the output signal of the probe under different scanning angles; (**b**) comparison of experiment and simulation of sensitivity change caused by probe scanning angle.

Firstly, the orthogonal axial probe and the conventional circular probe of different sizes are wound, and the sensitivity comparison test of the orthogonal axial probe and the conventional circular probe, is carried out. In Table 3: the sensitivity to the detection of weld seam defects in planar structures of the orthogonal axial probe and conventional circular probe of the similar size shown in the Table were found to be very close using the test of different defects of ordinary steel plates. The relationship between the deflection angle of the orthogonal axial probe and the probe sensitivity was further studied through experiments based on the scanning method of the orthogonal axial probe obtained by simulation. Under the conditions of 2 mm lift-off, 2.0 V excitation voltage, and 130 kHz excitation frequency, the orthogonal axial probe was used to scan the weld seam defects of the carbon steel weld seam test block at different angles. Figure 15 represents a waveform diagram with deflection angles 0° and 30° when scanning for defect as 25 × 0.2 × 1.0 mm. The relationship between the scanning angles and the sensitivity of the probe was obtained through quantitative calculation.

This study showed some deviation between the actual sensitivity value of the orthogonal axial probe and the simulation result. However, its relationship with the scanning angle was basically consistent with the change trend in the simulation results. The sensitivity of the probe first increased with the increase in the scan deflection angle. When the deflection angle was greater than 30°, it decreased with the increase in the angle. That is, when the probe deflection angle was 30°, the orthogonal axial probe obtained the best detection effect, which was consistent with the simulation results.

### 3.2. Experiment of Probe to Suppress Lift-off Effect

**Figure 16:**
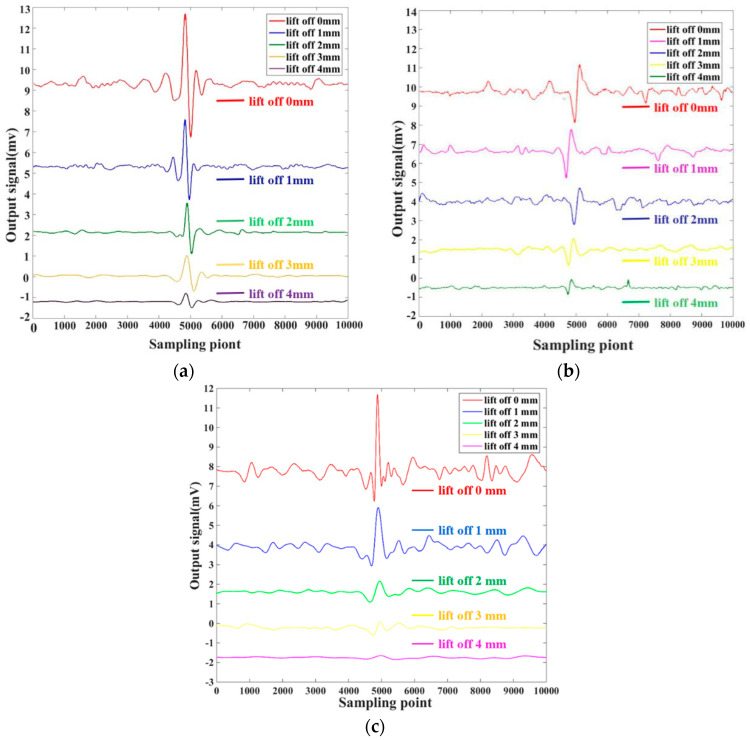
Different probes scan the output signal of the weld under different lift-off values: (**a**) orthogonal axial probe; (**b**) conventional circular probe; (**c**) conventional orthogonal probe.

Theoretical and simulation studies showed that the orthogonal axial probe obviously suppressed the lift-off effect caused by the unevenness of the surface of the test piece. Combined with the aforementioned results of the scanning angle of the detection probe, the defect scanning experiment was carried out with different lift-off value of 0, 1, 2, 3, and 4 mm. Between the probe and the test specimen under the condition of the excitation voltage of 2.0 V and the excitation frequency of 130 kHz, the waveform diagram was obtained in Figure 16a. This study found that the orthogonal axial probe designed in this study had different suppression effects for different lift-off value. When the lift-off value was less than 2 mm, the effective signal was already effectively determined, although the lift-off effect caused by the unevenness of the surface of the test piece was still obvious. When the lift-off value was between 2 and 4 mm, it was still difficult for the conventional circular probe to determine the effective signal of the defect, and this probe also obviously suppressed the uneven lift-off effect of the test piece compared with the conventional circular probe. When the distance was greater than 4 mm, the effective signal was difficult to be detected, and the probe could not detect crack defects normally. Therefore, the optimal detection lift-off value of the orthogonal axial probe was between 2 and 4 mm.

Under the same excitation conditions, same number of turns, and height, a circular probe with a diameter of 3 mm, conventional orthogonal probe and an orthogonal axial probe were tested and compared. Figure 16 shows the waveform comparison chart when scanning for weld crack defects as 25 × 0.2 × 1.0 mm, and the lift-off value was 0–4 mm. According to research and experiments, the orthogonal axial probe was found to suppress the lift-off effect, which the conventional circular probe lacked in any lift-off situation due to the unevenness of the weld seam area. The scan results in Figure 16 show that when the lift-off value was 1 mm, the signal-to-noise ratio of the detection signal of the orthogonal axial probe was much higher than that of the conventional circular probe. The maximum noise peak value of the orthogonal axial probe was about 13% of the peak value of the defect signal, and the weld seam defect signal was obvious and easy to distinguish. The noise peak of the conventional circular probe was about 68% of the peak of the defect signal. The noise was obvious, and the effective signal of the defect could not be determined. When the lift-off value was 3 mm, the conventional circular probe had basically lost the ability to detect weld seam defects. The ratio of the peak value of noise to the peak of the defect signal was about 56%, and the defect and the noise signal could not be discriminated. On the contrary, the suppression of the orthogonal axial probe on the lift-off effect was very obvious. The ratio of the maximum noise peak to the defect signal was about 6%, which could well detect the crack defect in the weld seam. Compared with the conventional orthogonal probe, the signal-to-noise ratio of the orthogonal axial probe is lower than that of the conventional orthogonal probe under the same lift-off value. When the lift-off value is 1 mm, the signal-to-noise ratio of the orthogonal axial probe is about 18% less than that of the conventional orthogonal probe and the output signal of the defect is about 21.4% larger than that of the conventional orthogonal probe. When the lift-off value is greater than 1 mm, due to the eddy current distribution of the conventional orthogonal probe being more concentrated on the probe surface, the weld defect output signal is very small, which makes the conventional orthogonal probe lose the reliability of detecting weld defects. On the contrary, the orthogonal axial probe designed by the author still has better signal-to-noise ratio and detection sensitivity. 

The experiment proved that the orthogonal axial probe significantly suppressed the lift-off effect caused by the unevenness of the weld seam surface, and has a high sensitivity in detecting weld defects, which was consistent with the theoretical and simulation results.

## 4. Conclusions

In the present study, a new type of orthogonal axial probe was proposed, and finite element simulation analysis and experiments on the orthogonal axial probe were carried out. The obtained findings of the study were as follows:

(1) The relationship between the dimensions and sensitivity of the orthogonal axial probe is studied by simulation, and an optimal probe size is obtained. When the length is 7 mm, the width is 5 mm, the height is 2 mm, and the number of turns is 200, the probe has the best sensitivity.

(2) The scanning mode of the orthogonal probe is studied through theory and simulation. When the scanning angle between the probe and the defect is 30°, the probe has the best defect scanning effect, and the output signal value was higher than that of parallel scanning of about 20%. Finally, the result was validated by experiment tests on a carbon steel plate.

(3) In the experiment of carbon steel plate weld defect detection, compared with the conventional circular probe, the orthogonal axial probe suppresses the lift-off effect caused by the uneven surface of the weld surface is much higher than that of the conventional circular probe. When the lift-off value of the probe is 1 mm, the signal-to-noise ratio of the orthogonal axial probe is 13%, while that of the conventional circular probe is 68%. When the lift-off of the probe is 3 mm, the signal-to-noise ratio of the orthogonal axial probe is 6%, while the conventional circular probe has basically lost the ability to detect weld defects. Compared with the conventional orthogonal probe. Under the same lift-off value, the orthogonal axial probe has better signal-to-noise ratio and detection sensitivity than the conventional orthogonal probe. When the lift-off value is 1 mm, the signal-to-noise ratio of the orthogonal axial probe is about 18% less than that of the conventional orthogonal probe and the output signal of the defect is about 21.4% larger than that of the conventional orthogonal probe. When the lift-off value is greater than 1mm, conventional orthogonal probes have lost the reliability of detecting weld defects, while orthogonal axial probes still have good signal-to-noise ratio and sensitivity.

Compared with the conventional circular probe and orthogonal probe, the orthogonal axial probe can suppress the lift-off effect caused by the uneven surface of the weld and has high sensitivity in the detection. It improved the reliability of the carbon steel plate weld defect detection of pressure equipment. However, it should be noted that, in this paper, the best scanning method of the probe is under the premise of knowing the direction of the defect; we cannot know the direction of the buried defects, so the probe function being improved to enable it to scan defects in any direction with good results is pending in future work. In addition, more work will be done to further validate and refine the probe performance, including the development of an array probe with this probe as a unit and performing more experiments to study the deep buried defects of the carbon steel weld.

## Figures and Tables

**Table 1:** Dimensions of the simulation model.

Parameter	Excitation Coil	Detection Coil	Air	Carbon Steel Plate	Weld Defect
Length (L/mm)	7	5		40	8
Width (H/mm)	5	3		40	2
Height (/mm)	2	2		4	0.2
Radius (R/mm)			40		

**Table 2:** Parameters and values of simulation material.

Material	Attributes	Conductivity (S/m)	Relative Permeability
Coil	copper	5.998 × 10^7^	1
Weld test specimen	iron	1.12 × 10^7^	4000
Air area	air	10	1

**Table 3:** Design size of Orthogonal axial probe.

Parameter	Excitation Coil	Detection Coil
Length (L/mm)	7	7
Width (H/mm)	5	5
Height (D/mm)	2	2
Turns (N)	200	200
